# Supplementary figures and images for: USP26 suppresses type I interferon signaling by targeting TRAF3 for deubiquitination
Source: PLoS One. 2024 Jul 26;19(7):e0307776. doi: 10.1371/journal.pone.0307776 (PMC11280224; doi:10.1371/journal.pone.0307776)

Fig 1C

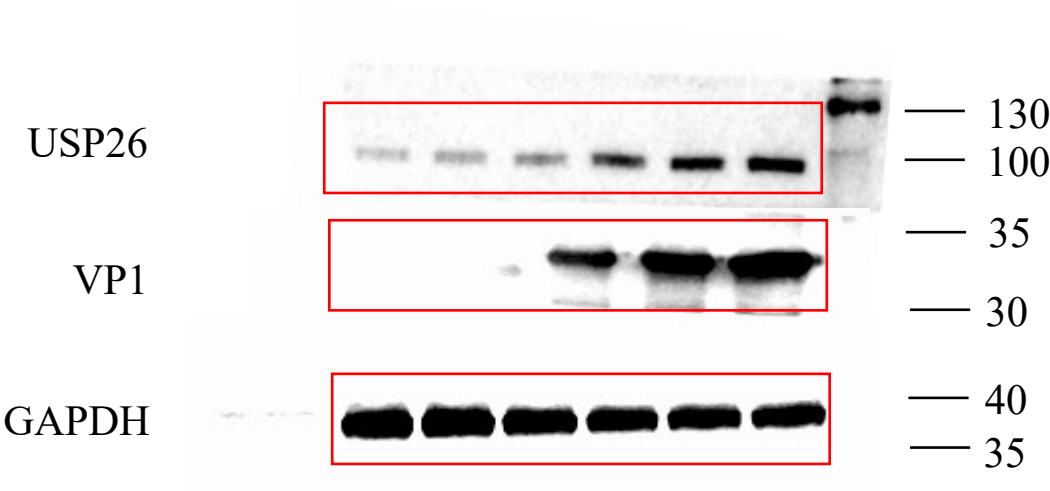

USP26

130

100

GAPDH

50

40

35

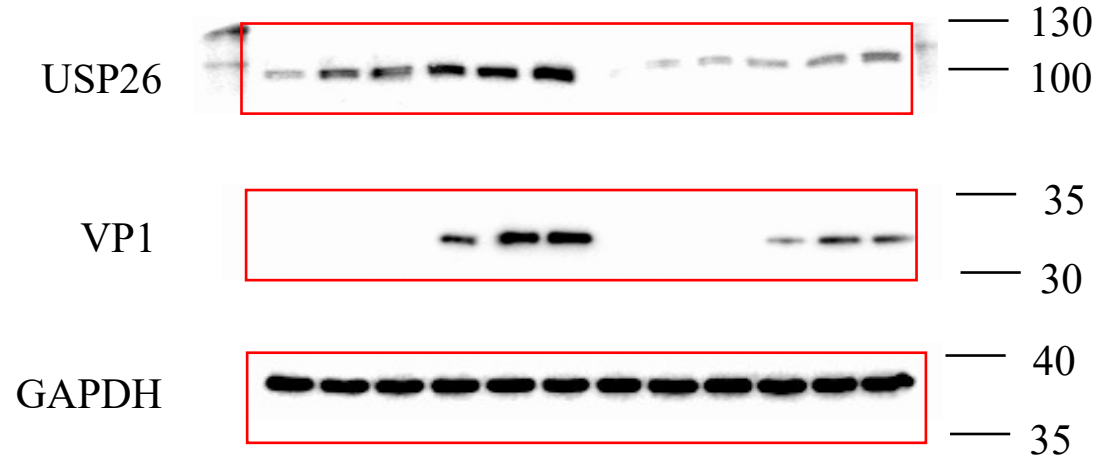

Fig 3I

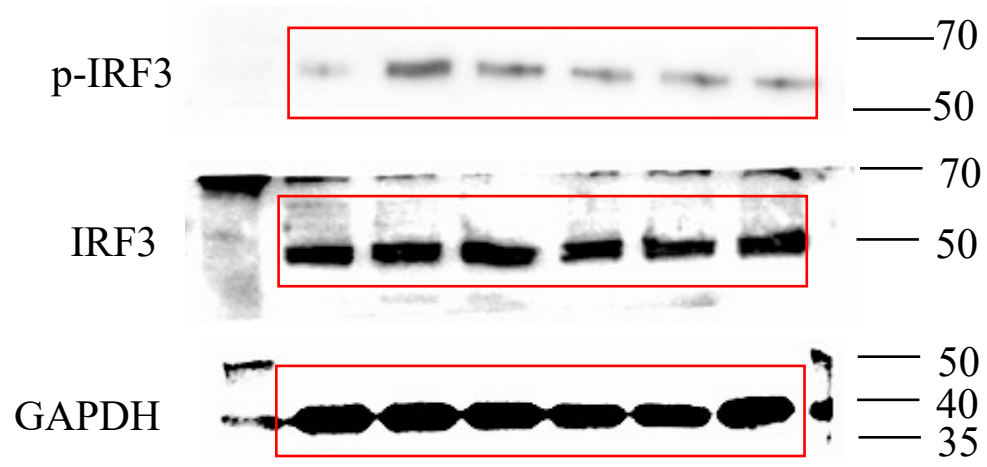

Fig 3J

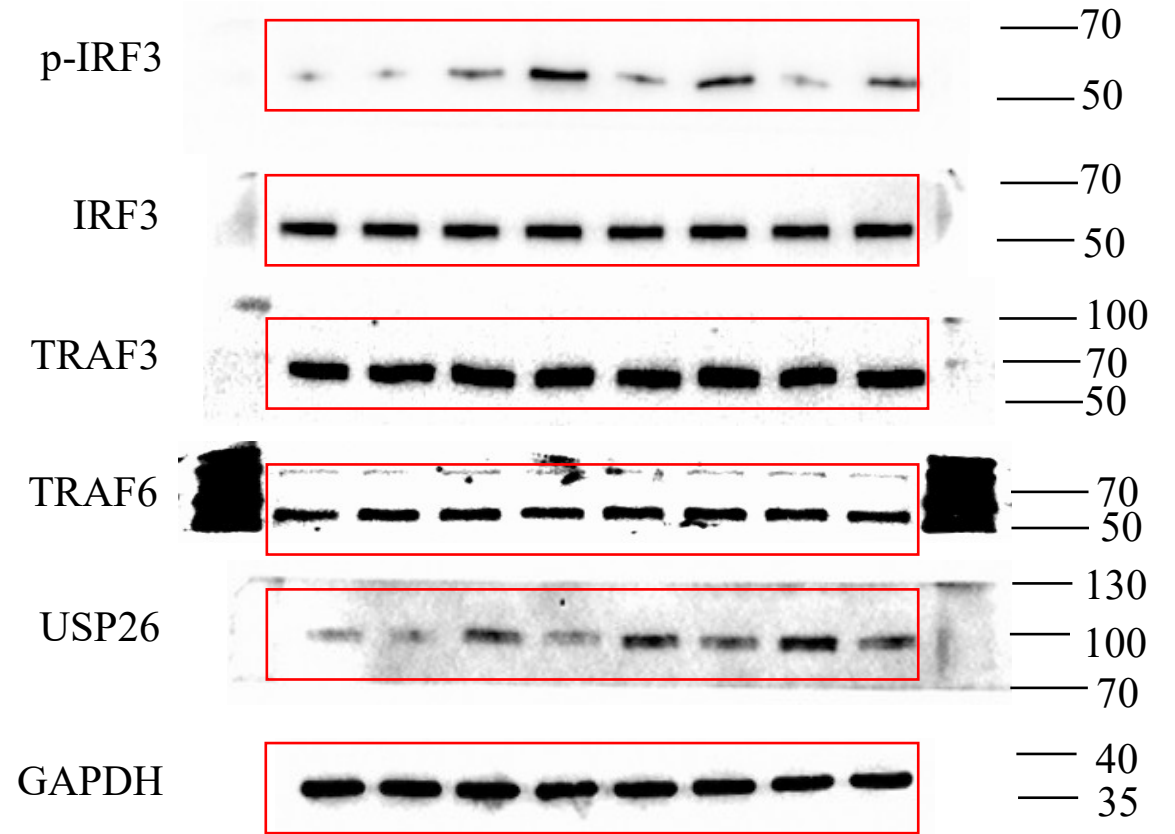

Fig 5A

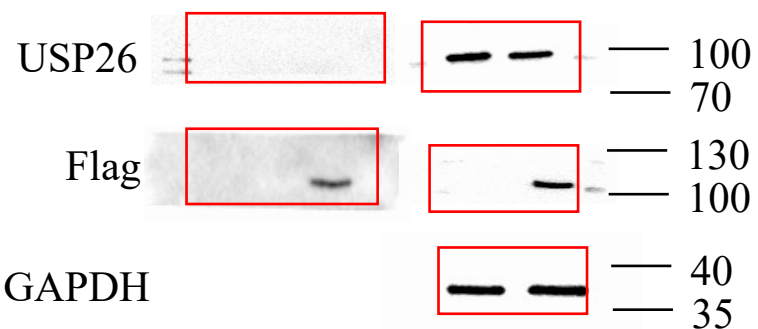

Fig 5B

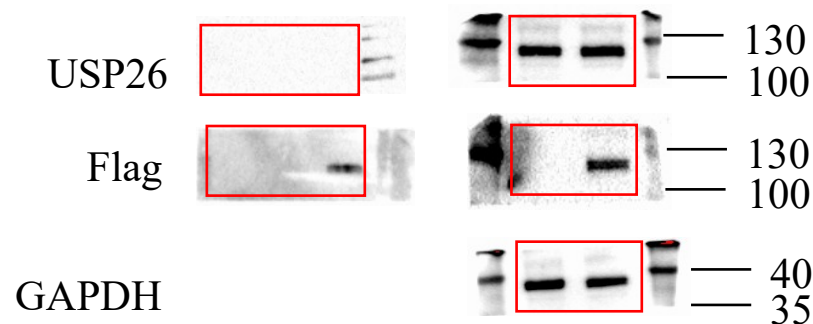

Fig 5C

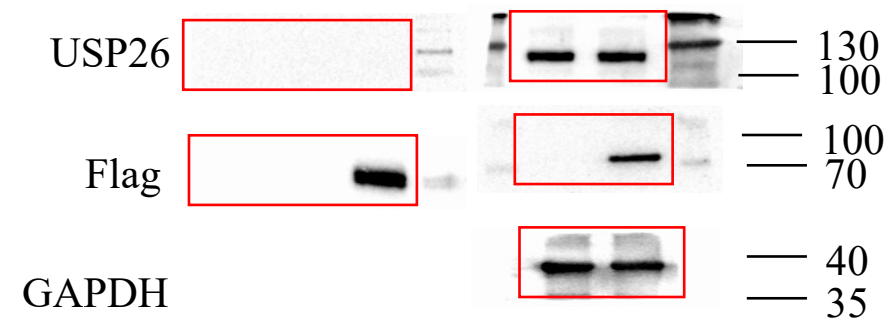

Fig 5D

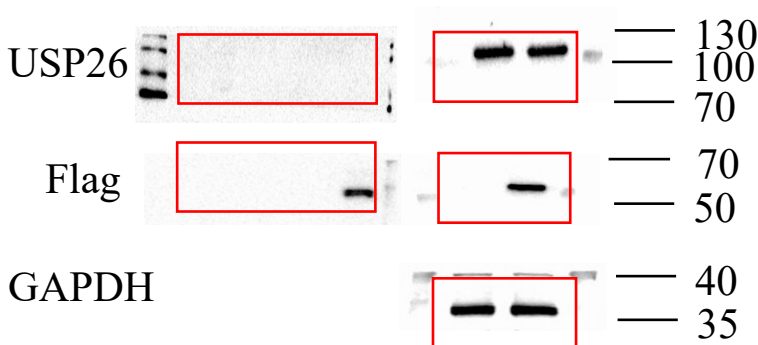

Fig 5E

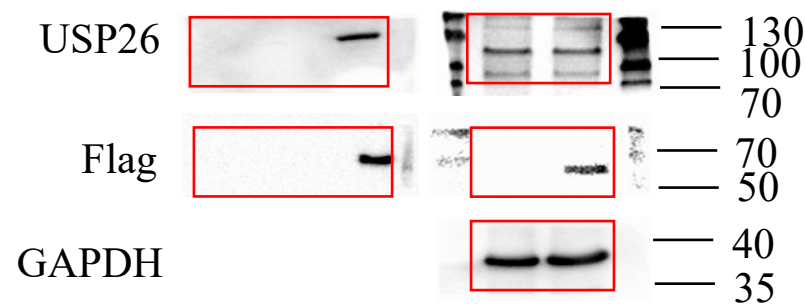

Fig 5F

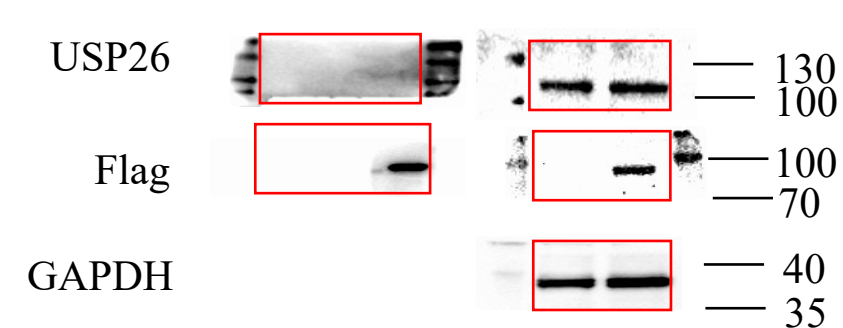

Fig 5G

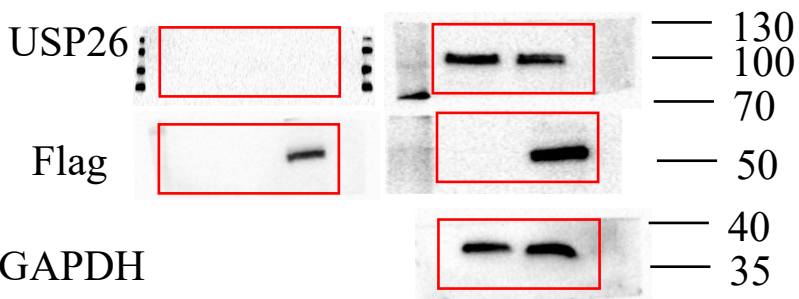

Fig 5H

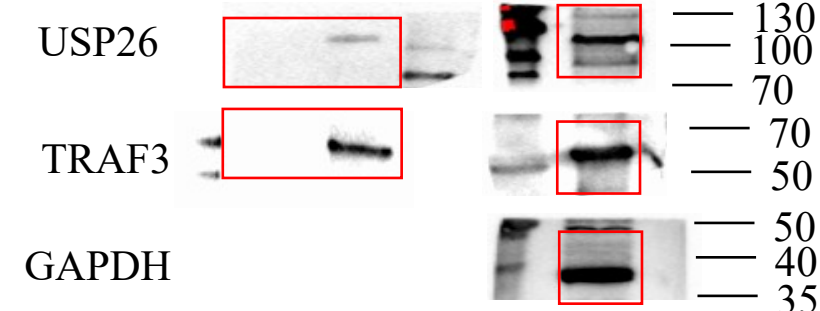

Fig 6A

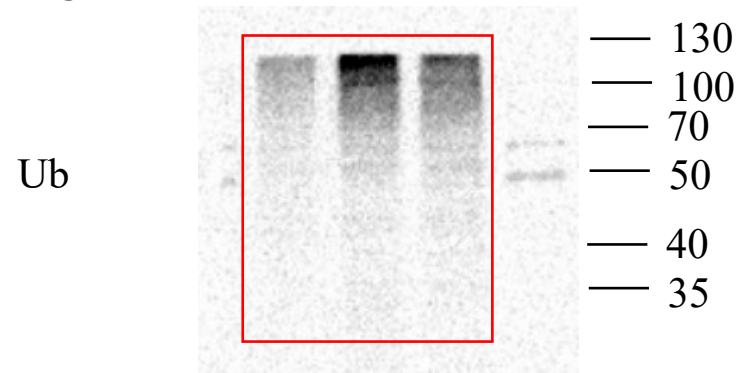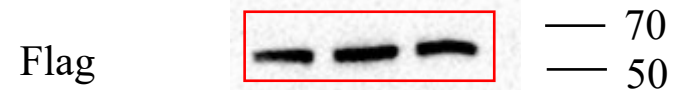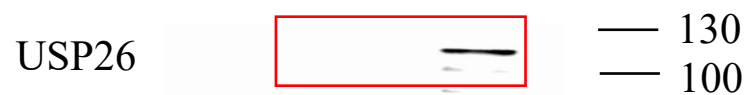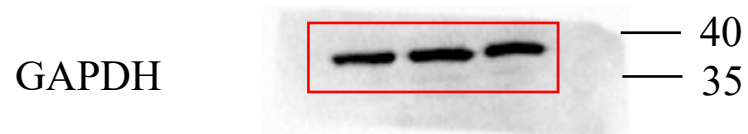

Fig 6B

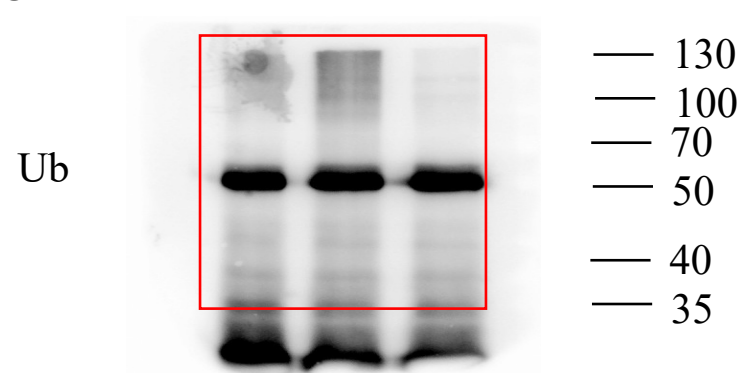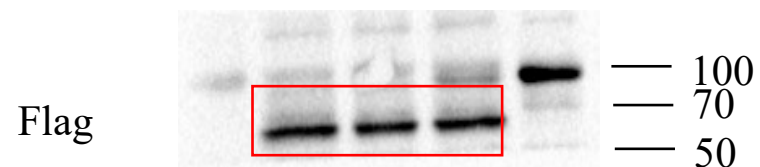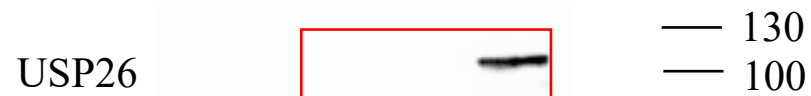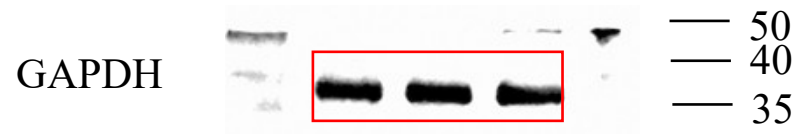

Fig 6C

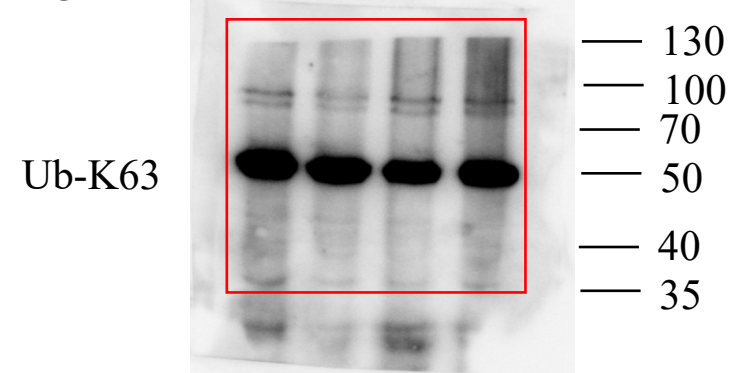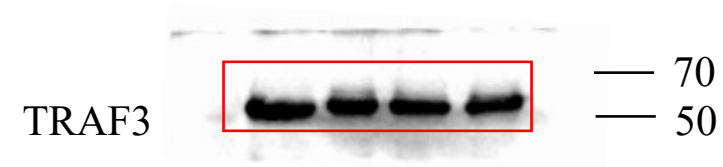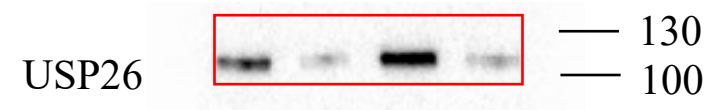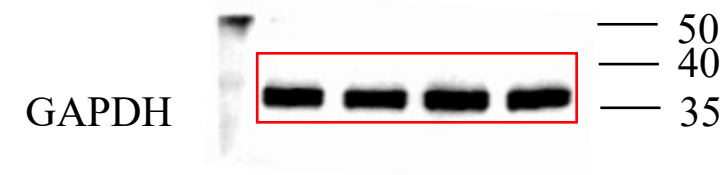

Supplement: S1 Raw images — (PDF) [file pone.0307776.s001.pdf]
