# Supplementary material for: USP26 suppresses type I interferon signaling by targeting TRAF3 for deubiquitination
Source: PLoS One. 2024 Jul 26;19(7):e0307776. doi: 10.1371/journal.pone.0307776 (PMC11280224; doi:10.1371/journal.pone.0307776)
Supplement: Attachment — Submitted filename: Response to Reviewers.docx [file pone.0307776.s002.docx]

Dear editor and reviewers:

We really appreciate you and reviewers for carefully reviewing our manuscript entitled **“USP26 suppresses type I interferon signaling by targeting TRAF3 for deubiquitination”** and giving us highly positive evaluation, helpful comments and constructive suggestions. Based on the reviewers’ helpful comments and constructive suggestions, we have revised the manuscript according to the reviewers’ suggestions. The point-to-point responses for your concerns were also enclosed in this letter. Revised portion are marked in red in the revised manuscript with track changes. We thank Doctor Jin-Hua Huang for editing this manuscript. We believe that the quality of the revised manuscript was greatly improved under the help of you and the reviewer. Meanwhile, we hope that the revised manuscript will be satisfactory and acceptable for the publication in **PLOS ONE*.*** The principal amendments to the paper and responses to the reviewers' comments are as follows:

**Responds to the editor comments**

1. Please ensure that your manuscript meets PLOS ONE's style requirements, including those for file naming.

**Response:** We have ensured that the revised manuscript meets PLOS ONE's style requirements, including those for file naming.

2. We suggest you thoroughly copyedit your manuscript for language usage, spelling, and grammar.

**Response:** We have thoroughly proofread the manuscript for language usage, spelling and grammar. Dr. Jin-Hua Huang has extensive experience in writing and has published articles with IF=17 (DOI: [10.1002/adfm.202212317](https://www.x-mol.com/paperRedirect/1601690639010484224)).

3. To comply with PLOS ONE submissions requirements, in your Methods section, please provide additional information regarding the experiments involving animals and ensure you have included details on (1) methods of sacrifice, (2) methods of anesthesia and/or analgesia, and (3) efforts to alleviate suffering.

**Response:** We have provided detailed information regarding the experiments involving animals in our Methods section. We have removed the ethics statement in the Methods section. If required, please state: " This study was approved by the Ethics Committee of Shanghai Tenth People’s Hospital of Tongji University (SHSY-IEC-4.1/19-120/01)."

4. Thank you for stating the following financial disclosure: “Financial support was provided by the Science Foundation of Shanghai Health Commission, China (20194Y0108)” Please state what role the funders took in the study.  If the funders had no role, please state: "The funders had no role in study design, data collection and analysis, decision to publish, or preparation of the manuscript." If this statement is not correct you must amend it as needed. Please include this amended Role of Funder statement in your cover letter; we will change the online submission form on your behalf.

**Response:** Financial support was provided by the Science Foundation of Shanghai Health Commission, China (20194Y0108). The funders had no role in study design, data collection and analysis, decision to publish, or preparation of the report.

5. Thank you for stating the following in the Acknowledgments Section of your manuscript: “Financial support was provided by the Science Foundation of Shanghai Health Commission, China (20194Y0108), Chongming science and technology support plan (CKY2022-43). The authors have no other relevant affiliations or financial involvement with any organization or entity.”

**Response:** We have removed funding-related text from the manuscript. Meanwhile, we've added new content.

6. When completing the data availability statement of the submission form, you indicated that you will make your data available on acceptance.

**Response:** We agree with your comments and decide on a data sharing plan before acceptance. All relevant data are within the paper and its [Supporting Information](https://journals.plos.org/plosone/article?id=10.1371/journal.pone.0298502#sec016) files.

7. PLOS ONE now requires that authors provide the original uncropped and unadjusted images underlying all blot or gel results reported in a submission’s figures or Supporting Information files.

**Response:** We have provided the original uncropped and unadjusted images underlying all blot or gel results reported in a submission’s figures or Supporting Information files.

8. We notice that your supplementary table is included in the manuscript file. Please remove them and upload them with the file type 'Supporting Information'. Please ensure that each Supporting Information file has a legend listed in the manuscript after the references list.

**Response:** We have removed them.

**Responds to the reviewer’s comments**

**Main** **Comment:** One question at this point is that why USP26 did not show up in the last publication of the same lab (PMID: 28391724). What was the coverage and replicability of the array study?

**Response:** Because we tested more than 80 deubiquitinating enzymes, only a few will be selectively chosen for publication in the article. PCR microarrays are only preliminary evidence of changes in deubiquitinating enzyme expression during EV71 infection., and other experimental methods are still needed for further demonstration.

**1.Comment:** Only when some experiments are provided can link USP26 to RLR signaling and TRAF3. It is possible that Poly I:C and EV71 can activate non-RLR signaling in RD cells, such as toll-like receptor signaling (although should be minimal? Should provide evidences or references). In addition, no evidence was provided to show siUSP26 promotes type I IFN production through TRAF3. For example, would siTRAF3 but not siTRAF6 abolish the IFN induction by siUSP26?

**Response:** Thank you very much for your comments. EV71 was suggested to trigger cellular anti-viral IFN-I signaling through MDA5, and Poly(I:C) of HMW has also been demonstrated as a ligand for MDA5-mediated type I IFN signaling activation ((DOI:10.1128/JVI.01080-07) and (DOI:10.1038/cr.2013.170)). As shown in Figure 3J, though the induction of USP26 by EV71 infection, stimulation of RD cells with Poly(I:C) did not induce significant upregulation of USP26. (Probably, Poly(I:C) does not show pathogenetic activity like EV71 virus and thus does not usurp USP26 for immune escape.) However, USP26 knockdown consistently increased the cellular sensitivity to HMW Poly(I:C)-induced IRF3 activation, which is in accord with the results observed for RD cells stimulated by EV71. Taken together, these results indicate that USP26 is a negative modulator of cellular type I IFN signaling. I hope these explanations clear up your confusion.

**2.Comment:** Figure 5F is suboptimal. A band is merely observed in IP/USP26 blot.

**Response:** Thank you very much for your suggestions. We have looked at the original image, there are no band present.

**3.Comment:** Figure 5G, input immunoblots have error. Flag-IRF3 should be expressed in one control only? Instead, USP26 was only found in the IRF3 overexpressed cells. Shall the immunoblots be reversed? It will be better to provide size indication to each band of the IP experiment.

**Response:** Thank you for your careful work. Due to our carelessness that led to this result, we have modified this figure.

**4.Comment:** Figure 6A-6C are suboptimal. Where is the mono-Ub on figure 6A? Moreover, please provide size for all immunoblots. Poly-Ub smear should appear above the unlinked protein.

**Response:** Thank you very much for your comments. We have provided size for all immunoblots. I am very sorry that I have not been able to meet your requirements. Ubiquitination modification experiments are affected by many factors, especially antibody quality. The unsatisfactory results of our experiments were mainly due to the limited laboratory conditions. We hope you will understand.

**5.** **Comment:** Line 247-249 is duplicated. The description to 3A-D was incomplete. Please revise.

**Response:** Thank you for your careful work. We have revised this paragraph.

**6. Comment:** Section 3.3, the use of the term ‘type I IFN signaling’ might be confusing. As the study focused on IRF3 activation, it is better to use ‘type I IFN production’ instead.

**Response:** Thank you for your valuable advice. We have revised this paragraph.

## Sincerely yours

Chao Xu

Department of Clinical Laboratory, Chongming Brach Shanghai Tenth People’s Hospital, Tongji University School of Medicine,

Shanghai 202150, P. R. China.

Tel: +86-021-59418281
E-mail: 18706190100@163.com
